# Validation of the PreOperative Score to predict Post-Operative Mortality (POSPOM) in Dutch non-cardiac surgery patients

**DOI:** 10.1186/s12871-022-01564-1

**Published:** 2022-03-03

**Authors:** Annick Stolze, Ewoudt M. W. van de Garde, Linda M. Posthuma, Markus W. Hollmann, Dianne de Korte-de Boer, Valérie M. Smit-Fun, Wolfgang F. F. A. Buhre, Christa Boer, Peter G. Noordzij, on behalf of the TRACE Study investigators, Wolfgang F. F. A. Buhre, Christa Boer, Dianne de Korte-de Boer, Annick Stolze, Linda M. Posthuma, Valérie M. Smit-Fun, Sander van Kuijk, Peter G. Noordzij, Myra Rinia, Jens-Peter Hering, Bas in’t Veld, Gert-Jan Scheffer, Carmen Dirksen, Marja Boermeester, Jaap Bonjer, Cees Dejong, Markus W. Hollmann

**Affiliations:** 1 Department of Anesthesiology, Amsterdam University Medical Centre, VU University Amsterdam, De Boelelaan 1117, 1081 HV Amsterdam, The Netherlands; 2 Department of Clinical Pharmacy, St. Antonius Hospital Nieuwegein, Nieuwegein, The Netherlands; 3 Department of Anesthesiology, Amsterdam University Medical Centre, University of Amsterdam, Amsterdam, The Netherlands; 4 Department of Anesthesiology and Pain Medicine, Maastricht University Medical Centre+, Maastricht, The Netherlands; 5 Department of Anesthesiology, Intensive Care and Pain management, St. Antonius Hospital Nieuwegein, Nieuwegein, The Netherlands

**Keywords:** Calibration, Complications, Discrimination, In-hospital mortality, Outcome, Peri-operative, Risk assessment, Validation

## Abstract

**Background:**

Standardized risk assessment tools can be used to identify patients at higher risk for postoperative complications and death. In this study, we validate the PreOperative Score to predict Post-Operative Mortality (POSPOM) for in-hospital mortality in a large cohort of non-cardiac surgery patients. In addition, the performance of POSPOM to predict postoperative complications was studied.

**Methods:**

Data from the control cohort of the TRACE (routine posTsuRgical Anesthesia visit to improve patient outComE) study was analysed. POSPOM scores for each patient were calculated post-hoc. Observed in-hospital mortality was compared with predicted mortality according to POSPOM. Discrimination was assessed by receiver operating characteristic curves with C-statistics for in-hospital mortality and postoperative complications. To describe the performance of POSPOM sensitivity, specificity, negative predictive values, and positive predictive values were calculated. For in-hospital mortality, calibration was assessed by a calibration plot.

**Results:**

In 2490 patients, the observed in-hospital mortality was 0.5%, compared to 1.3% as predicted by POSPOM. 27.1% of patients had at least one postoperative complication of which 22.4% had a major complication. For in-hospital mortality, POSPOM showed strong discrimination with a C-statistic of 0.86 (95% CI, 0.78–0.93). For the prediction of complications, the discrimination was poor to fair depending on the severity of the complication. The calibration plot showed poor calibration of POSPOM with an overestimation of in-hospital mortality.

**Conclusion:**

Despite the strong discriminatory performance, POSPOM showed poor calibration with an overestimation of in-hospital mortality. Performance of POSPOM for the prediction of any postoperative complication was poor but improved according to severity.

## Introduction

Postoperative mortality has decreased significantly over the last few decades. The European Surgical Outcomes Study (EuSOS) reported an overall in-hospital mortality of 4% in patients undergoing non-cardiac surgery across 28 European nations in 2011 [1]. More specifically for the Dutch surgical population, an all-cause mortality rate of 1.85% was reported for major surgery performed during 1991–2005 [2]. The TRACE investigators published a 30-day mortality of 0.5% in major non-cardiac surgery patients in the period 2016–2018 [3].

Current improvements in perioperative care aim to reduce *failure to rescue*, thus lowering the proportion of preventable deaths due to unnoticed complications in the postoperative period [4]. Besides death, postoperative complications are associated with adverse functional outcome after surgery. The challenge is to improve postoperative survival and prevent new disability, especially in selected groups of high risk patients. Standardized risk assessment tools can be used to distinguish patients at higher risk for postoperative complications and death, and are recommended by international societies for perioperative risk stratification [5].

Various risk assessment tools for the prediction of adverse postoperative outcome have been published [6]. In 2016, Le Manach and others developed and validated the PreOperative Score to predict Post-Operative Mortality (POSPOM) [7]. A strength of POSPOM is that it is based on a large cohort consisting of more than 5.5 million patients who underwent heterogeneous surgical procedures originating from the French National Hospital Discharge Data Base (NHDBB), which makes it representative for the European surgical population.

The performance of the POSPOM score has been evaluated mostly in specific surgical populations, including patients undergoing emergency abdominal surgery, [8] radical cystectomy, [9] vascular surgery, [10, 11] and elderly patients with hip fractures [12]. Two studies have evaluated POSPOM in large heterogeneous surgical populations, using single center retrospective data [13, 14]. However, both studies did not address the performance of POSPOM in terms of sensitivity, specificity, negative predictive values (NPV) and positive predictive values (PPV), which limits the clinical applicability.

In this study we validated the POSPOM score in a heterogeneous Dutch cohort of nearly 2500 non-cardiac surgery patients by using data from the control cohort of the multicenter stepped-wedge cluster randomized interventional TRACE (routine posTsuRgical Anesthesia visit to improve patient outComE) study [3]. In addition, we studied the potential of the POSPOM score to predict the development of postoperative complications. The aim of the study was to investigate to what extent the POSPOM score is able to predict in-hospital mortality and major postoperative complications in the TRACE cohort.

## Methods

### Study design and participants

This study was based on the TRACE (routine posTsuRgical Anesthesia visit to improve patient outComE) study. Details on design and analysis of the TRACE study have been previously reported [15]. TRACE was a prospective, multicenter, stepped-wedge, cluster-randomized interventional study performed between October 2016 and August 2019 in nine academic and non-academic hospitals in The Netherlands. The effects of a routine postoperative visit by an anesthesiologist on the incidence of postoperative complications and mortality was assessed. For the current study, participants that originated from the control cohort (non-intervention) of the TRACE study were included. The study was ethically approved by the Human Subjects Committee of Amsterdam UMC, location VUmc Amsterdam (number NL56004.029.16, 29-06-2016) and registered with the Netherlands Trial Register (NTR5506). The Clinical Research Unit of the Amsterdam UMC took responsibility for the monitoring of patient inclusion and data registration. All study participants signed informed consent.

### Study data and variables

The POSPOM score has been originally developed by Le Manach and others and is composed of a set of preoperative variables including age, medical history and type of surgery [7]. For each variable a certain number of points is assigned to the final score, which varies between 0 and 70 points and equates to a probability of in-hospital mortality. To calculate a POSPOM score for each patient, relevant preoperative variables were extracted from the TRACE database.

Type of surgery was defined in the original TRACE database, however the list of procedures was less detailed compared to POSPOM. Therefore appropriate modifications were made in the surgical classification of POSPOM to calculate the POSPOM score for all study patients. First, because of the in- and exclusion criteria of the TRACE study, day case procedures and cardiac and orthopedic trauma surgery were not included. Due to the absence of day case procedures, the POSPOM surgery groups ‘other orthopedic’ and ‘minor gastrointestinal’ were not represented in the study cohort. Second, within TRACE no subdivision was made between minor and major surgery. Hence, for urologic, vascular and hepatic procedures this subdivision was based on duration of surgery; > 120 min was considered as major surgery for urologic and vascular procedures and > 180 min for hepatic procedures.

### Study endpoints

The primary study endpoint was in-hospital mortality. The secondary study endpoints were the occurrence of any complication, severity of a complication graded according to the Clavien-Dindo classification, [16] and type of complication (i.e. infectious, cardiac/transfusion, pulmonary, venous thromboembolic, renal, neurological, surgical, ileus, delirium and other).

### Statistical analysis

The size of the cohort was determined within the sample size calculation of the TRACE study of which detailed descriptions have been published previously [15]. Continuous variables were expressed as mean ± standard deviation (SD) and in case of a non-symmetric distribution as median with interquartile range (IQR). Categorical variables were described with frequencies and percentages. For the calculation of the discriminating power of POSPOM, receiver operating characteristic (ROC) curves with corresponding C-statistics (i.e. area under the curve (AUC) values) were calculated for primary and secondary study endpoints [17]. For the description of the performance of the POSPOM score sensitivity, specificity, negative predictive values (NPV), and positive predictive values (PPV) were calculated from true-positive, false-positive, true-negative, and false-negative values for the outcome variables in-hospital mortality and major complication (Clavien-Dindo grade III-V). To assess the mean calibration (or calibration-in-the-large) the average predicted risk of in-hospital mortality was compared with the overall observed rate of in-hospital mortality [18]. In addition, the difference between predicted and observed in-hospital mortality rates per POSPOM score group were calculated. A calibration plot for in-hospital mortality was constructed with corresponding calibration curve intercept and slope; the predicted probability was based on the average POSPOM score per tentile. All analyses were performed using SPSS Statistics version 26.0 (IBM, Armonk, NY, USA) and GraphPad Prism version 8.2.1 (GraphPad Software, San Diego, California, USA).

## Results

### Study population

In total, 5473 patients were included in the TRACE study cohort. After removal of 283 drop-outs (i.e. withdrawal of informed consent before operation, operation cancelled and/or not meeting inclusion criteria) and 2700 patients originating from the intervention cohort, 2490 patients were included in the analysis. Median age was 65 (IQR 12) years, 46.9% of patients were female, and a majority (61.7%) was classified as ASA class 2. Active cancer or diabetes mellitus were the most common comorbidities. Major gastrointestinal surgery was the most frequently performed procedure, followed by arthroplasty and spine surgery, and minor urologic surgery (Table 1).

**Table 1:** Study characteristics

Demographics	Value	Missing (n)	POSPOM points
Total cohort	2490 (100)		
Age (year) (median, IQR)	65 (12)		≥21y: per 5y + 1
Female	1169 (46.9)		N/A
**Comorbidities**			
Cardiac arrhythmia	275 (11)		+ 1
Cerebrovascular disease	181 (7.3)	1	+ 1
COPD	228 (9.2)	1	+ 1
Diabetes mellitus	381 (15.3)	1	+ 1
Ischemic heart disease	291 (11.7)		+ 1
Peripheral vascular disease or abdominal aortic aneurysm	225 (9)		+ 1
Preoperative chronic dialysis	27 (1.1)	1	+ 1
Chronic renal failure	189 (7.6)	2	+ 2
Dementia	11 (0.4)		+ 2
Transplanted organs	26 (1)	2	+ 2
Chronic respiratory failure	61 (2.4)	2	+ 3
Active cancer	945 (38)	1	+ 4
Chronic alcohol abuse	45 (1.8)	3	+ 4
Chronic heart failure or cardiomyopathy	96 (3.9)	2	+ 4
Hemiplegia, paraplegia or paralytic syndrome	33 (1.3)	1	+ 4
**Type of surgery**^a^			
Endoscopic digestive	55 (2.2)		+ 0
Ophtalmologic	4 (0.2)		+ 0
Gynecologic	221 (8.9)		+ 6
Interventional cardiorhythmology	2 (0.1)		+ 6
Arthroplasty and spine^b^	477 (19.2)		+ 8
Ear, nose and throat	76 (3.1)		+ 9
Minor urologic^b^	238 (9.6)		+ 9
Plastic and reconstructive	54 (2.2)		+ 9
Major urologic^b^	196 (7.9)		+ 9
Other surgery	17 (0.7)		+ 12
Minor hepatic	11 (0.4)		+ 12
Renal transplant	22 (0.9)		+ 12
Minor vascular^b^	34 (1.4)		+ 13
Major hepatic	55 (2.2)		+ 13
Thoracic	108 (4.3)		+ 13
Neurosurgery	121 (4.9)		+ 14
Major vascular^b^	132 (5.3)		+ 16
Major gastrointestinal^b^	647 (26)		+ 16
Interventional neuroradiology	1 (0)		+ 17
Transplant	12 (0.5)		+ 22
Multiple trauma related	7 (0.3)		+ 22

Data represent frequencies (%), unless otherwise stated

^a^Some patients may have had more than 1 type of surgery

^b^Marked surgery subgroups differ from the original POSPOM type of surgery classification. See method section for a clarification of the modifications

*Abbreviations*: *POSPOM* PreOperative Score to predict Post-Operative Mortality, *IQR* interquartile range, *N/A* not applicable, *COPD* Chronic Obstructive Pulmonary Disease

### Incidence of mortality and postoperative complications

**Fig. 1:**
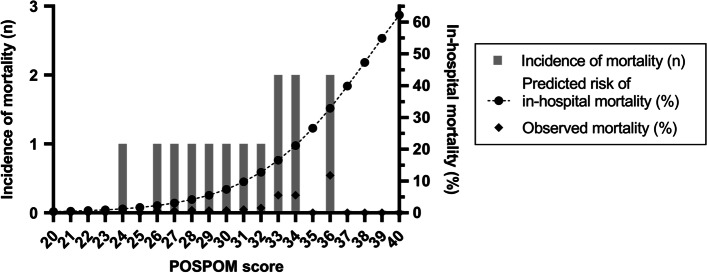
Distribution of patients with mortality among POSPOM scores. *No deaths were observed below a POSPOM score of 24 points and above a POSPOM score of 36 points.* Abbreviations: *POSPOM *PreOperative Score to predict PostOperative Mortality

The distribution of in-hospital mortality according to POSPOM score is presented in Fig. 1. Based on the median POSPOM score of 24 (IQR 8) points, the predicted risk of in-hospital mortality was 1.3% in our study population. The observed in-hospital mortality rate was 0.5% (14 patients). Of these patients POSPOM scores varied between 24 and 36 points, which equates a probability of in-hospital mortality of 1.3 and 32.9% respectively. POSPOM values less than 24 points were associated with 0% observed mortality (Fig. 1).

**Fig. 2:**
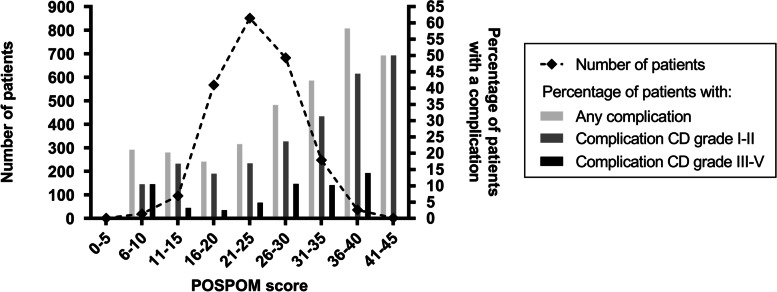
Distribution of patients with complications among POSPOM scores. *Complications are classified according to the Clavien-Dindo classification of surgical complications.* Abbreviations: *POSPOM *PreOperative Score to predict PostOperative Mortality, *CD* Clavien-Dindo

Six hundred and seventy-six patients (27.1%) had at least one postoperative complication. A majority (39.3%) of complications was scored Clavien-Dindo (CD) grade II at highest (requiring pharmacological treatment), followed by 33.4% CD grade I (any deviation from the normal postoperative course, without the need for pharmacological treatment or surgical, endoscopic and radiological interventions) and 15.7% CD grade III (requiring surgical, endoscopic or radiological intervention). Forty-two patients (6.2%) had a life-threating complication requiring ICU-management. The distribution of the observed in-hospital complication rates among POSPOM scores is presented in Fig. 2. Of all postoperative complications, the incidence of other complications (not further defined) was highest (45.1%), followed by infectious (36.5%) and cardiac/transfusion (27.5%).

### Validation of POSPOM for mortality and postoperative complications

#### Discrimination

**Fig. 3:**
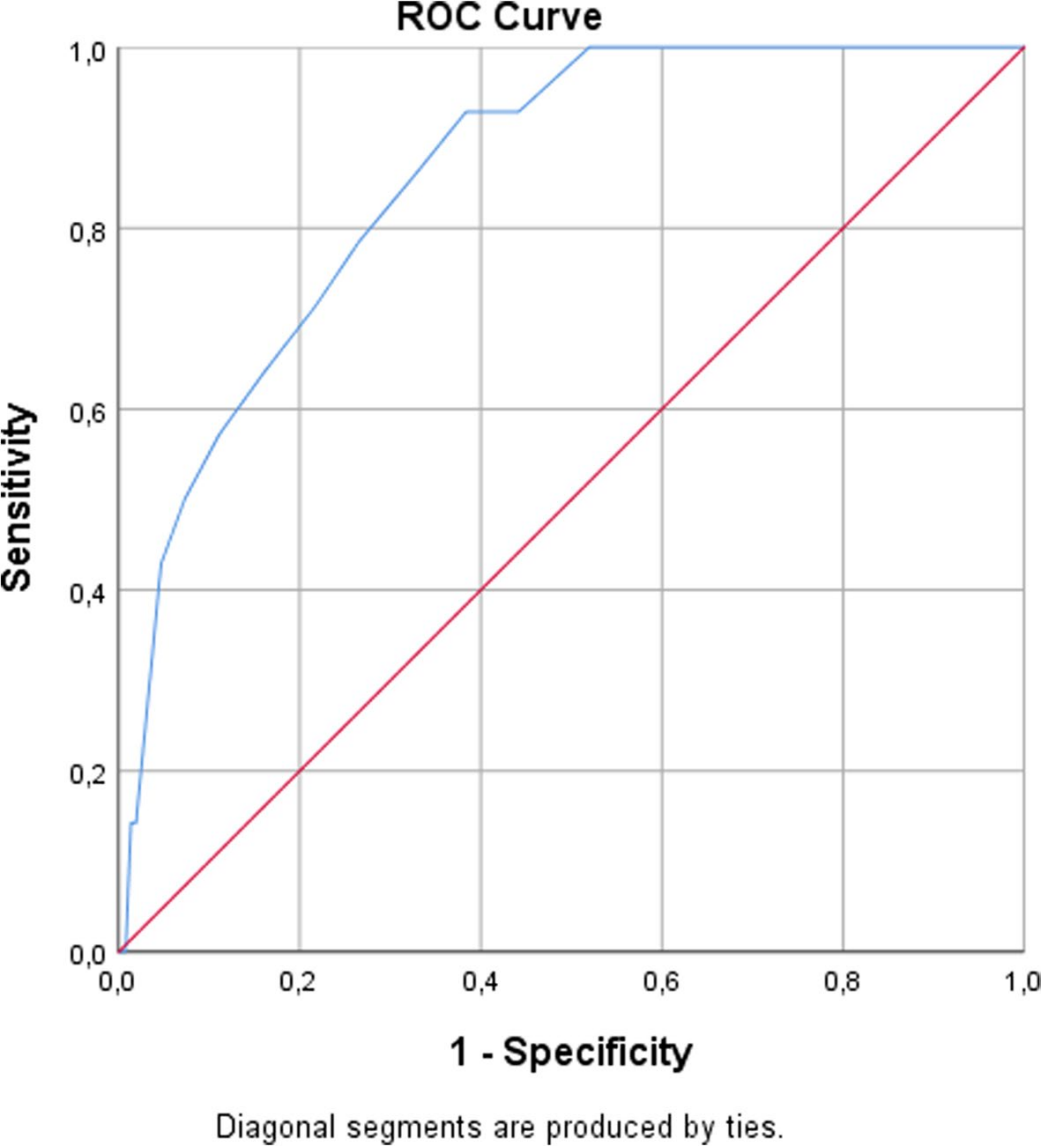
ROC curve: predicted versus observed in-hospital mortality for POSPOM. *ROC curve with a corresponding C-statistic of 0.86 (95% CI, 0.78–0.93), which implies strong discriminating power.* Abbreviations: *ROC* Receiver Operating Characteristics, *POSPOM*  PreOperative Score to predict PostOperative Mortality

The concordance between POSPOM calculated mortality and observed mortality was strong (C-statistic 0.86 (95% CI, 0.78–0.93). The corresponding Receiver Operating Characteristics (ROC) curve is shown in Fig. 3. A cutoff value of a POSPOM score of 24 points equated to a sensitivity of 100%, a specificity of 48%, a PPV of 1.1% and an NPV of 100%. Despite a strong C-statistic for postoperative mortality, the low incidence of in-hospital mortality resulted in low PPV and high NPV values. Other cutoff values of the POSPOM score did not result in improvement of the PPV (Table 2).

**Table 2:** Performance of different POSPOM cutoff values for the prediction of mortality

POSPOM score ≥	Sensitivity	Specificity	NPV	PPV	Number of patients (n)	Deaths (n)
24	100.0	48.0	100.0	1.1	1298	14
26	92.9	61.6	99.9	1.4	961	13
28	78.6	73.4	99.8	1.6	668	11
30	64.3	83.8	99.8	2.2	410	9
32	50.0	92.7	99.7	3.7	187	7
34	28.6	96.7	99.6	4.7	86	4
36	14.3	98.6	99.5	5.6	36	2
38	0.0	99.5	99.4	0.0	13	0

*Abbreviations*: *POSPOM* PreOperative Score to predict PostOperative Mortality, *NPV* negative predictive value, *PPV* positive predictive value

**Fig. 4:**
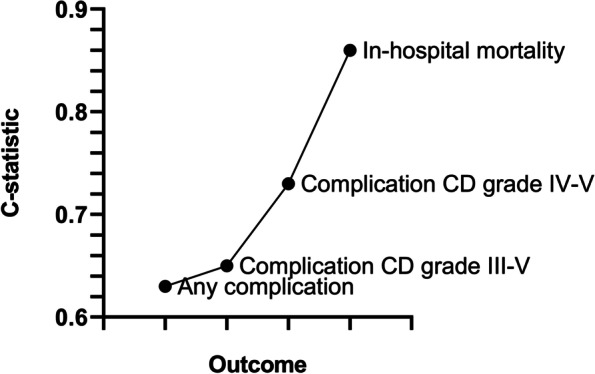
C-statistic values for different outcomes. *Complications are classified according to the Clavien-Dindo classification of surgical complications.* Abbreviations: *CD *Clavien-Dindo

The overall discriminatory ability of POSPOM for any postoperative complication was poor (C-statistic 0.63 (95% CI, 0.60–0.65)) but improved according to severity (C-statistic 0.65 (95% CI, 0.61–0.70) for CD grade III-V and C-statistic 0.73 (95% CI, 0.67–0.80) for CD grade IV-V; Fig. 4). For different types of complications, the discrimination was lowest for neurological complications (C-statistic 0.54 (95% CI, 0.40–0.68)) and highest for pulmonary complications (C-statistic 0.73 (95% CI, 0.69–0.78)). For the prediction of major complications, the cutoff value of 38 points showed the highest PPV of 20.0%, nevertheless with a corresponding high NPV of 93.5% (Table 3).

**Table 3:** Performance of different POSPOM cutoff values for the prediction of major complication

POSPOM score ≥	Sensitivity	Specificity	NPV	PPV	Number of patients (n)	Major complication (CD grade III-V) (n)
6	100.0	0.0	100.0	6.6	2463	162
8	99.4	0.1	75.0	6.5	2460	161
10	99.4	0.4	90.0	6.6	2454	161
12	98.8	1.3	93.9	6.6	2431	160
14	98.8	2.3	96.4	6.6	2408	160
16	96.9	4.7	95.6	6.7	2350	157
18	95.7	8.6	96.6	6.9	2259	155
20	90.1	21.0	96.8	7.4	1964	146
22	85.8	34.9	97.2	8.5	1638	139
24	74.1	49.2	96.4	9.3	1290	120
26	63.0	62.9	96.0	10.7	956	102
28	45.1	74.2	95.1	11.0	666	73
30	28.4	84.2	94.4	11.2	409	46
32	14.2	92.8	93.9	12.2	188	23
34	7.4	96.7	93.7	13.6	88	12
35	3.1	98.0	93.5	9.6	52	5
36	3.1	98.6	93.5	13.2	38	5
38	1.9	99.5	93.5	20.0	15	3
40	0.6	99.7	93.4	12.5	8	1
42	0.0	100.0	93.4	0.0	1	0

*Abbreviations*: *POSPOM* PreOperative Score to predict PostOperative Mortality, *NPV* negative predictive value, *PPV* positive predictive value, *CD* Clavien-Dindo

#### Calibration

**Fig. 5:**
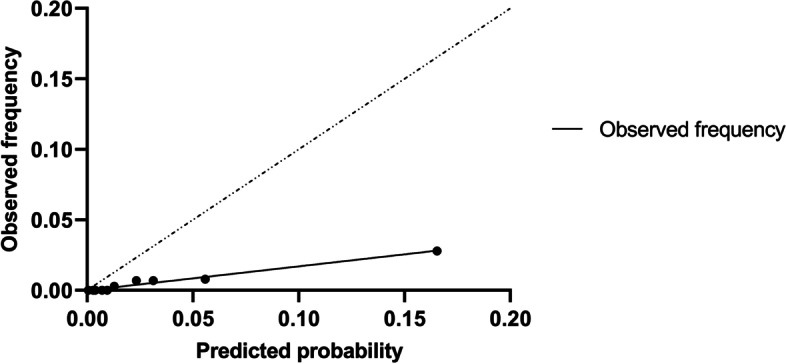
Calibration plot for in-hospital mortality. *The slope of the fitted line is 0.1704 and the y-axis intercept is 0.00 (95% CI, − 0.001395-0.001345)*

The average predicted risk of in-hospital mortality was higher than the overall observed mortality rate, which indicated that POSPOM overestimated risk in general (‘calibration-in-the-large’). In addition, for each particular POSPOM score group the predicted risk of in-hospital mortality exceeded the observed percentage of in-hospital mortality (Table 4 in Appendix), especially in the groups with higher POSPOM score. The calibration plot for in-hospital mortality (Fig. 5) showed poor calibration of POSPOM with a fitting curve slope of 0.17 and a negative y-axis interception, which suggests too extreme estimated risks and overestimation respectively.

## Discussion

In this large cohort of Dutch non-cardiac surgery patients, we found a strong discrimination for the prediction of in-hospital mortality with POSPOM. This suggests that POSPOM scores are capable to rank patients from low to high risk for in-hospital mortality during their perioperative course [17]. On the other hand, the poor agreement between predicted and observed in-hospital mortality rates indicated poor calibration; i.e. POSPOM systematically overestimated the risk of in-hospital mortality in general [18]. For the prediction of complications, the discrimination of POSPOM was poor to fair according to the severity of the complication.

The strong C-statistic (> 0.8) of POSPOM for the prediction of in-hospital mortality found in this surgical cohort is in line with two previously published validation studies [8, 9]. Two studies reported a mean observed mortality rate (‘calibration-in-the-large’) lower than predicted by POSPOM which is in accordance with our results [9, 12]. One study found an observed mortality similar to predicted [11]. Of all preceding validation studies, two studies examined calibration more closely via a calibration plot [8, 14]. Both studies found underestimation of in-hospital mortality in the POSPOM risk groups with a low risk of in-hospital mortality, whereas in the higher risk groups POSPOM overestimated mortality. In our patients, an overestimation of in-hospital mortality for all POSPOM groups was observed. The subgroup of patients with high POSPOM scores were overrepresented compared to the derivation cohort of Le Manach and others, nevertheless the observed mortality rate of 0.5% in the TRACE cohort was comparable with the mortality rate of 0.47% in the derivation cohort. An explanation for this finding may be the decrease in surgery-related risks over the years; patients in the original POSPOM cohort underwent surgery in 2010 compared to 2016 until 2018 in the TRACE cohort.

The low incidence of postoperative in-hospital death resulted in a clinically irrelevant low PPV for the prediction of mortality. For the prediction of a major complication, the PPV values were homogenous for the majority of the POSPOM cutoff value groups, which resulted in poor differentiation for the prediction of a major complication. The highest PPV of 20% was found for the POSPOM cutoff value of 38 points. Nevertheless, because of the low number of patients in this subgroup, the use of this cutoff value to distinguish patients at higher risk will not be of added value in clinical practice.

Our study was limited by the low absolute number of deaths which negatively influences the quality of the validation of the POSPOM score in this cohort. Validation of the POSPOM score in a population with a higher a priori chance of a complicated postoperative course may have led to different results. As described in the method section, unavoidable modifications of the surgical classification were made which may have influenced the calculation of the final POSPOM score. In addition, cardiac and orthopedic trauma patients were absent in our cohort. Strengths of the TRACE control cohort are its prospective and multicenter design, large size and heterogeneous surgery patient group.

In our study cohort the performance of the POSPOM score to predict in-hospital mortality and complications was poor. In a population with a higher a priori chance of a complicated postoperative hospital stay, using POSPOM as a standardized risk assessment tool may be of added value. However, despite all the risk models that have been developed in the last couple of decades, there is a lack of evidence with regard to the clinical consequences of a higher predicted risk of in-hospital death or complications. The role of these risk models in clinical practice has still to be determined. Besides, because of the low mortality rates in current European healthcare, it may be more interesting to focus on the prediction of complications and relevant outcomes which lead to disability instead of mortality.

## Conclusion

In this study POSPOM showed strong discrimination for the prediction of in-hospital mortality, however the calibration of POSPOM was poor. In addition, for the prediction of complications the performance of POSPOM was poor. This limits the use of the risk score in clinical practice.

## Data Availability

The datasets used and/or analysed during the current study are available from the corresponding author on reasonable request.

## Appendix

**Table 4:** Observed and predicted risks of in-hospital mortality for different POSPOM scores

POSPOM score ≥	Incidence of mortality (n)	Number of patients (n)	Observed risk of in-hospital mortality (%)	Predicted risk of in-hospital mortality (%)	Difference between predicted and observed in-hospital mortality (%)
20	0	174	0.0	0.4	0.4
21	0	159	0.0	0.5	0.5
22	0	189	0.0	0.7	0.7
23	0	162	0.0	1.0	1.0
24	1	195	0.5	1.3	0.8
25	0	143	0.0	1.7	1.7
26	1	144	0.7	2.3	1.6
27	1	149	0.7	3.1	2.5
28	1	120	0.8	4.2	3.4
29	1	138	0.7	5.6	4.9
30	1	128	0.8	7.4	6.6
31	1	95	1.1	9.8	8.7
32	1	65	1.5	12.8	11.2
33	2	36	5.6	16.5	11.0
34	2	36	5.6	21.1	15.6
35	0	14	0.0	26.6	26.6
36	2	17	11.8	32.9	21.2
37	0	6	0.0	39.9	39.9
38	0	3	0.0	47.3	47.3
39	0	4	0.0	54.9	54.9
40	0	6	0.0	62.2	62.2

*Abbreviations*: *POSPOM* PreOperative Score to predict PostOperative Mortality

## Abbreviations

AUC: Area under the curve
CD: Clavien-Dindo
CI: Confidence interval
COPD: Chronic obstructive pulmonary disease
EuSOS: The European Surgical Outcomes Study
IQR: Interquartile range
NHDBB: The French National Hospital Discharge Data Base
NPV: Negative predictive value
POSPOM: PreOperative Score to predict Post-Operative Mortality
PPV: Positive predictive value
ROC: Receiver operating characteristic
SD: Standard deviation
TRACE: Routine posTsuRgical Anesthesia visit to improve patient outComE
